# Facile Synthesis of CeO_2_-LaFeO_3_ Perovskite Composite and Its Application for 4-(Methylnitrosamino)-1-(3-Pyridyl)-1-Butanone (NNK) Degradation

**DOI:** 10.3390/ma9050326

**Published:** 2016-04-29

**Authors:** Kaixuan Wang, Helin Niu, Jingshuai Chen, Jiming Song, Changjie Mao, Shengyi Zhang, Saijing Zheng, Baizhan Liu, Changle Chen

**Affiliations:** 1 Anhui Province Key Laboratory of Environment-Friendly Polymer Materials, School of Chemistry and Chemical Engineering, Anhui University, Hefei 230601, China; wangkaixuan55@163.com (K.W.); cjshuai@126.com (J.C.); songjm@ustc.edu.cn (J.S.); maocj2011@gmail.com (C.M.); syzhangi@126.com (S.Z.); 2 Key Laboratory of Cigarette Smoke Research of China National Tobacco Corporation, Shanghai 200082, China; zhengsj@sh.tobacco.com.cn (S.Z.); liubz@sh.tobacco.com.cn (B.L.); 3 CAS Key Laboratory of Soft Matter Chemistry, Department of Polymer Science and Engineering, University of Science and Technology of China, Hefei 230026, China

**Keywords:** CeO_2_-LaFeO_3_, NNK, degradation, perovskite

## Abstract

A facile and environmentally friendly surface-ion adsorption method using CeCO_3_OH@C as template was demonstrated to synthesize CeO_2_-LaFeO_3_ perovskite composite material. The obtained composite was characterized by X-ray diffraction (XRD), fourier transform infrared spectra (FT-IR), field-emission scanning electron microscopy (FE-SEM), transmission electron microscopy (TEM), thermo-gravimetric analysis and differential scanning calorimetry (TG-DSC), N_2_ adsorption/desorption isotherms and X-ray photoelectron spectra (XPS) measurements. The catalytic degradation of nitrosamine 4-(methylnitrosamino)-1-(3-pyridyl)-1-butanone (NNK) was tested to evaluate catalytic activity of the CeO_2_-LaFeO_3_ composite. Much better activity was observed for the CeO_2_-LaFeO_3_ composite comparing with CeO_2_ and LaFeO_3_. These results suggested that perovskite composite materials are a promising candidate for the degradation of tobacco-specific nitrosamines (TSNAs).

## 1. Introduction

Perovskite materials can be described as ABO_3_, with A and B being two cations and O being an oxygen anion. Perovskite materials are well known for their advantageous physical and chemical properties for various applications such as catalysts [1,2], multiferroic materials [3], energy conversion materials [4,5], and gas sensors [6]. Recently, LaFeO_3_ and related materials have received increasing attention [7,8]. CeO_2_ has been extensively studied as a catalyst to transform some environmentally harmful substances into environmentally friendly materials, such as oxidation CO [9], selective reduction of NO [10] and photocatalytic degradation of Rhodamine B [11]. Therefore, it is highly fascinating to combine the advantages of LaFeO_3_ and CeO_2_ materials [12,13]. A variety of techniques have been developed to fabricate the CeO_2_-LaFeO_3_ composite materials, such as the solid-state reaction method [14], solution combustion and co-precipitation method [15], low-temperature thermal decomposition method [16] and ethylene diamine tetraacetic acid-citrate method [17]. In this work, we reported a facile and environmentally friendly surface-ion adsorption method to prepare perovskite-type LaFeO_3_ and a CeO_2_-LaFeO_3_ composite. The formation mechanism was also investigated.

Tobacco smoke contains more than 5000 kinds of compounds. Among these chemicals, tobacco-specific nitrosamines (TSNAs) play a significant role in causing lung cancer and other diseases for people who either use tobacco products or are exposed to secondhand smoke [18]. There are mainly four kinds of tobacco-specific nitrosamines: 4-(methylnitrosamino)-1-(3-pyridyl)-1-butanone (NNK), N’-nitrosonornicotine (NNN), N’-nitrosoanatabine (NAT) and N’-nitrosoanabasine (NAB) [19], of which NNK has been identified as the most potent lung carcinogen [20,21]. In order to protect public health, it is necessary to reduce the level of NNK in tobacco. Control of the storage environment, reduction of leaf nitrate contents, and the scavenging of gaseous nitrosating agents could be especially effective to reduce or inhibit NNK formation during the storage of cured tobacco [22]. Zeolites were successfully applied to capture and degrade NNK in gas or liquid phases [19,23]. Nanostructured titanates were also used as catalysts for selectively reducing NNK in mainstream cigarette smoke [24]. These results suggest that it is possible to efficiently degrade NNK contents with suitable catalysts. Since all TSNA materials contain N-NO group, it was hypothesized that the catalytic breaking of the chemical bond in the N-NO group by perovskite materials may be the key step for the decomposition of these carcinogens [19]. In this work, we preformed systematic studies on the properties of CeO_2_-LaFeO_3_ composite for the degradation of NNK.

## 2. Results and Discussion

**Figure 1:**
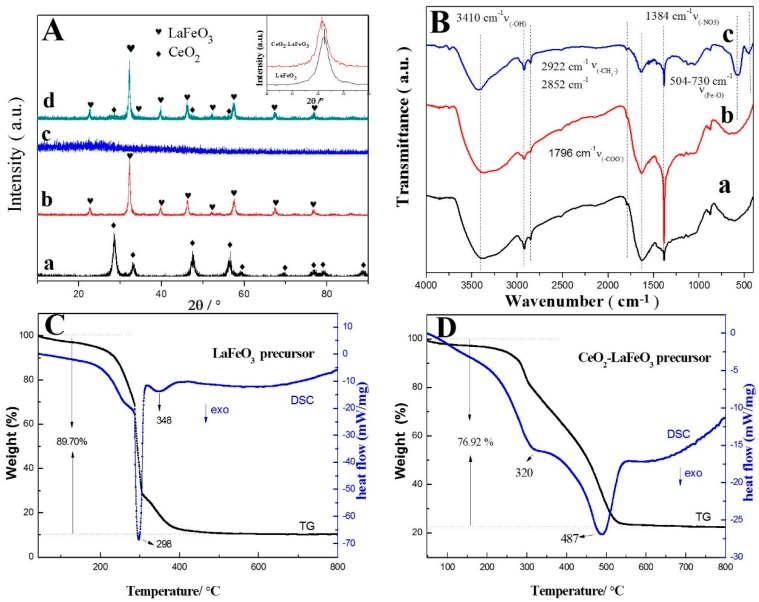
(**A**) XRD spectra of different samples: (**a**) CeO_2_; (**b**) LaFeO_3_; (**c**) CeO_2_-LaFeO_3_ precursor; and (**d**) CeO_2_-LaFeO_3_; Inset: X-ray diffraction peak around 32° of LaFeO_3_ and CeO_2_-LaFeO_3_; (**B**) FT-IR spectra of: (**a**) CeCO_3_OH@C; (**b**) CeO_2_-LaFeO_3_ precursor; and (**c**) CeO_2_-LaFeO_3_; TG-DSC curves of (**C**) LaFeO_3_ precursor; (**D**) CeO_2_-LaFeO_3_ precursor.

Figure 1A shows the X-ray powder diffraction (XRD) patterns of (a) CeO_2_; (b) LaFeO_3_; (c) CeO_2_-LaFeO_3_ precursor; and (d) CeO_2_-LaFeO_3_. The samples were scanned from 2θ degrees of 20° to 90°, using a Cu Kα radiation with a characteristic wavelength (λ) of 0.15405 nm. As shown in Figure 1A-a, the main characteristic peaks located at 2θ = 28.55°, 47.48°, 56.34° are corresponding to the (111), (220), (311) planes of CeO_2_, which are in good agreement with the reported XRD data (JCPDS Card No. 43-1002). All of these peaks are corresponding to a face-centered cubic (fcc) fluorite structure CeO_2_ [25]. Figure 1A-b can be assigned to the orthorhombic perovskite LaFeO_3_ structure with the Pbnm space group, and the diffraction peaks completely agree with those of JCPDS Card No. 37-1493. No obvious peak could be observed in the XRD pattern of CeO_2_-LaFeO_3_ precursor (Figure 1A-c), indicating that neither LaFeO_3_ nor CeO_2_ were formed after ultrasonic-assisted surface-ion adsorption treatment. In Figure 1A-d, the main diffraction peaks at 2θ = 22.61°, 32.19°, 39.67°, 46.14° and 57.39° belong to the LaFeO_3_ orthorhombic perovskite phase and the weak signals at 2θ = 28.55°, 47.48°, 56.34° correspond to the CeO_2_ phase concurrent with the major perovskite phase. These results indicate the formation of a CeO_2_-LaFeO_3_ composite. The main diffraction peak of the obtained CeO_2_-LaFeO_3_ was almost the same as that of LaFeO_3_. However, the peak slightly shifted to a lower angle (inset of Figure 1A), indicating an enlarged dimension of unit cell caused by the cerium substitution [26]. The crystallite size of the catalysts was estimated using Scherrer’s method as follows [27]:

D = Kλ/βcosθ
(1)

where D is the average diameter of the calculated particles; K is the shape factor of the average grain size (the expected shape factor is 0.89), λ is the wavelength characteristic in Å (in this particular case λ = 1.5405 Å); and β is the width of the X-ray peak at half height. The average crystallite size of CeO_2_, LaFeO_3_ and CeO_2_-LaFeO_3_ samples was found to be 12, 24 and 17 nm, respectively. In the CeO_2_-LaFeO_3_ composite, the diffraction peak for the CeO_2_ phase is extremely weak. Therefore, the average crystallite size in the CeO_2_-LaFeO_3_ composite was determined using the main reflections (2θ = 22.61°, 32.19°) corresponding to the LaFeO_3_ phase.

The FT-IR spectra of CeCO_3_OH@C, CeO_2_-LaFeO_3_ precursor and CeO_2_-LaFeO_3_ are shown in Figure 1B. The absorption peak centered at *ca*. 3410 cm^−1^ was attributed to the structural O-H stretching vibration modes of physically absorbed H_2_O on the samples or the surface O-H group in the materials, whereas the absorption peaks at *ca*. 2922 cm^−1^ and *ca.* 2852 cm^−1^ were attributed to C-H asymmetric and symmetric stretching vibrations. The absorption peaks at 1796 cm^−1^ were attributed to (COO^−^). The absorption peaks at *ca.* 1628 cm^−1^ (Figure 1B-a,b) were attributed to C=C vibrations [28,29], which became weaker after calcination (Figure 1B-c). The absorption peaks at *ca*. 1384 cm^−1^ were attributed to -CH_3_ symmetrical deformation vibration (Figure 1B-a,c). The peak being stronger (Figure 1B-b) was due to stretching vibration of nitrate anion adsorbed on the CeO_2_-LaFeO_3_ precursor surface, which overlapped with -CH_3_ symmetrical deformation vibration. The peak becoming weaker (Figure 1B-c *versus* Figure 1B-b) was ascribed to the decomposing of nitrate anions. The bands in the 504−730 cm^−1^ region (Figure 1B-c) were assigned to the stretching modes of the octahedral FeO_6_ groups in perovskite, which were the most important absorption bands of the perovskite structure [30]. The band located at *ca*. 453 cm^−1^ (Figure 1B-c) can be assigned to the deformation modes of the same polyhedra [31]. However, no peaks of CeO_2_ were detected. Based on the FT-IR analysis, it can be concluded that CeCO_3_OH@C possesses hydrophilic negatively charged groups such as -OH and -COO^−^ on its carbonaceous shell, and these groups can cause adsorption metal cations in aqueous solution. Through the subsequent calcination process, the carbonaceous shell could be eliminated and the composite formed. Therefore, CeCO_3_OH@C can be used as a template to synthesize perovskite composites.

The TG-DSC curves of LaFeO_3_ precursor and CeO_2_-LaFeO_3_ precursor were shown in Figure 1C,D. The total weight loss of LaFeO_3_ precursor (Figure 1C) was 89.70%, indicating a 10.30% LaFeO_3_ product yield. The sharp exothermic peak at 298 °C in the DSC curve corresponds to the heat generated by the burning of the carbonaceous spheres’ template and decomposing of nitrate anions [32]. The broad exothermic peak at about 348 °C can be attributed to the crystallization process of LaFeO_3_ [33]. The total weight loss of the as-prepared CeO_2_-LaFeO_3_ precursor was 76.92%. The broad exothermic peak at about 320 °C and the sharp exothermic peak at about 487 °C correspond to the combustion of carbon-rich polysaccharide (GCP) and formation of CeO_2_-LaFeO_3_.

**Figure 2:**
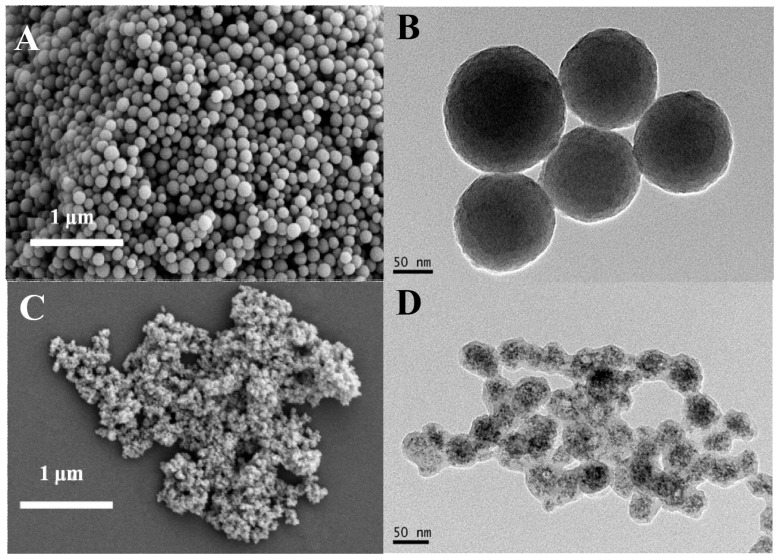
SEM and TEM images of (**A**,**B**) carbonaceous spheres; (**C**,**D**) CeCO_3_OH@C.

**Figure 3:**
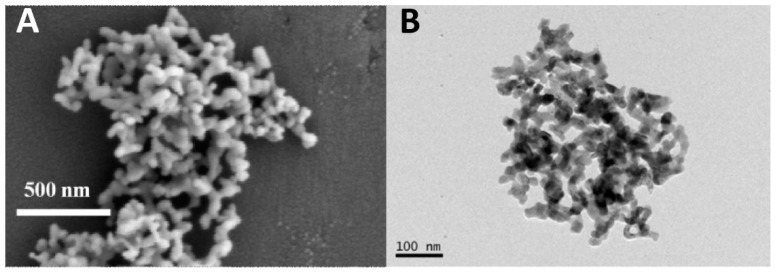

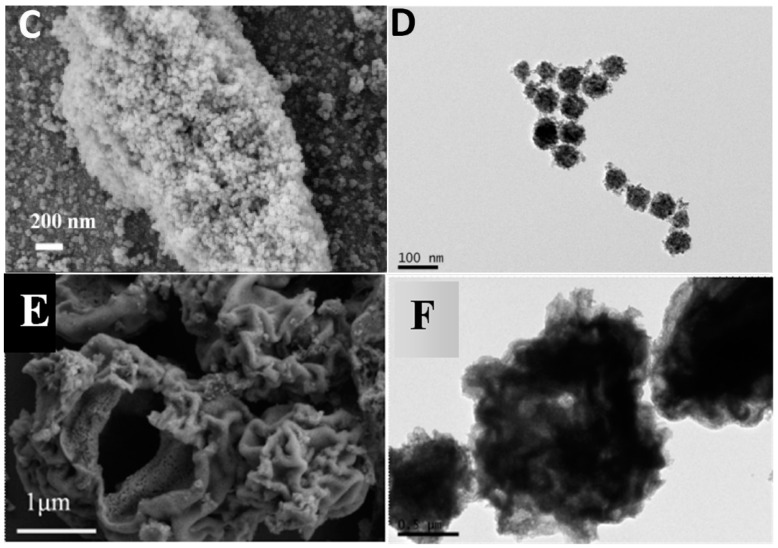
SEM and TEM images of (**A**,**B**) LaFeO_3_; (**C**,**D**) CeO_2_; (**E**,**F**) CeO_2_-LaFeO_3_.

The representative SEM and TEM images of the carbonaceous spheres and CeCO_3_OH@C templates are shown in Figure 2. After hydrothermal treatment, carbonaceous spheres formed and displayed good monodispersed spherical morphology with an average diameter of 150 nm (Figure 2A,B). Figure 2C,D are the SEM and TEM images of CeCO_3_OH@C. The GCP-coated CeCO_3_OH nanospheres were well retained. Figure 3D shows that CeCO_3_OH@C has a CeCO_3_OH core; a GCP shell has been prepared by the hydrothermal method. To some degree, the structure of GCP-coated CeCO_3_OH nanospheres have overlapped with the shell, which is a similar result to our previous work [27].

The representative SEM and TEM images of LaFeO_3_, CeO_2_ and CeO_2_-LaFeO_3_ are shown in Figure 3. The SEM image of LaFeO_3_ (Figure 3A) obtained by calcining the LaFeO_3_ precursor possesses a small branch-shape structure which consists of many small nanorods. The TEM result also indicates that the LaFeO_3_ (Figure 3B) nanostructure was stacking by smaller LaFeO_3_ nanorods with an average diameter of 24 nm (calculated by using Scherrer equation). In the SEM image (Figure 3D), CeO_2_ nanoparticles exhibit uniform size distribution and spherical morphology. The TEM image (Figure 3D) indicated that the sample is composed of tiny nanocrystallites (about 12 nm, calculated by using the Scherrer equation). The SEM image of CeO_2_-LaFeO_3_ (Figure 3E) displays an irregular porous structure. The morphology of CeO_2_-LaFeO_3_ also displays an irregular porous structure in the TEM image (Figure 3F).

**Figure 4:**
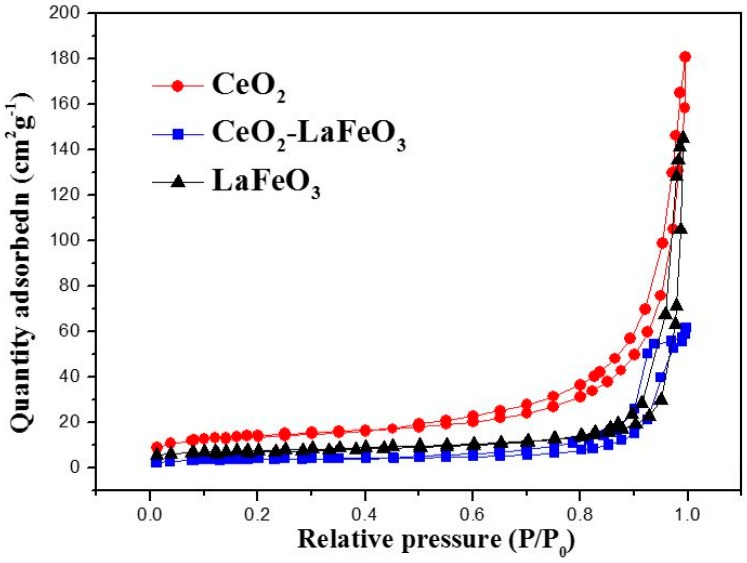
N_2_ adsorption and desorption isotherms of LaFeO_3_, CeO_2_ and CeO_2_-LaFeO_3_ composite.

Brunauer-Emmett-Teller (BET) analysis was used to investigate the specific surface area of the samples. The specific surface areas of CeO_2_, LaFeO_3_ and CeO_2_-LaFeO_3_ are 47.3, 24.7 and 12.7 m^2^·g^−1^, respectively. The N_2_ isotherm of CeO_2_-LaFeO_3_ shown in Figure 4 is close to Type IV (according to International Union of Pure and Applied Chemistry classification) with a hysteresis loop observed in the range of 0.4–1.0 *p*/*p*_0_, indicating the mesoporous structure of the CeO_2_-LaFeO_3_ composite.

**Figure 5:**
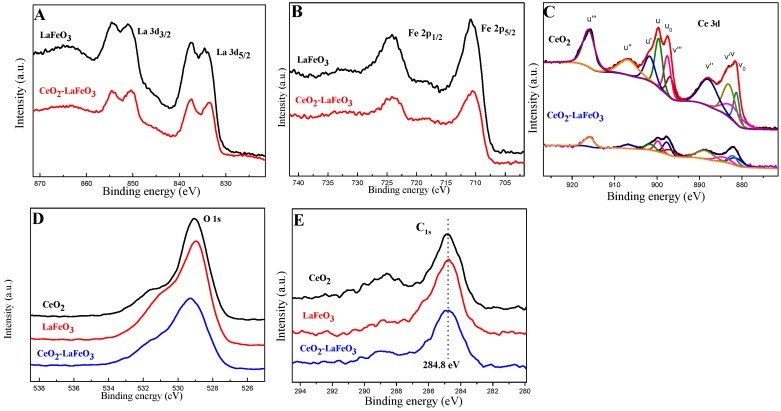
XPS spectra of (**A**) La 3d; (**B**) Fe 2p; (**C**) Ce 3d; (**D**) O 1s for LaFeO_3_, CeO_2_ and CeO_2_-LaFeO_3_; (**E**) C 1s.

The oxidation states of principal elements of LaFeO_3_ were analyzed by XPS. All the binding energy values obtained in the XPS analysis were calibrated using C 1s (284.8 eV) as the reference. Figure 5A–E display the XPS spectra of La 3d_5/2_, Fe 2p, Ce 3d and O 1s and C 1s core levels for the as-prepared CeO_2_, LaFeO_3_ and CeO_2_-LaFeO_3_. The peaks of La 3d_5/2_ and La 3d_3/2_ for LaFeO_3_ in Figure 5A are situated at 833.9 and 837.5 eV and at 851.1 and 854.6 eV, while the peaks of La 3d_5/2_ and La 3d_3/2_ for CeO_2_-LaFeO_3_ in Figure 5A are situated at 833.6 and 837.4 eV and at 850.42 and 854.5 eV. These results confirm the presence of La^3+^ ions [34]. In Figure 5B, the peaks at 710.8 and 724.1 eV of LaFeO_3_ are attributed to the binding energies of Fe 2p_3/2_ and 2p_1/2_, while the peaks at 710.6 and 724.2 eV of CeO_2_-LaFeO_3_ are attributed to the binding energies of Fe 2p_3/2_ and 2p_1/2_, respectively. No noticeable shoulder peaks are found in the Fe 2p XPS spectrum, indicating that Fe ions are in the Fe^3+^ oxidation state [35]. The Ce 3d transition peaks of CeO_2_ and CeO_2_-LaFeO_3_ composites are shown in Figure 5C. The v_0_, v’, u_0_, and u’ peaks are attributed to Ce^3+^, whereas v, v’’, v’’’, u, u’’, and u’’’ are attributed to Ce^4+^. The Ce^4+^/Ce^3+^ atomic ratio has been obtained from the area of the peaks obtained by the deconvolution procedure [36]. In this way, a Ce^3+^/(Ce^4+^ + Ce^3+^) ratio of 0.32 has been obtained on the CeO_2_ sample, while the amount of Ce^3+^ species increases on the CeO_2_-LaFeO_3_ composite (Ce^3+^/(Ce^4+^ + Ce^3+^) atomic ratio of 0.39). Especially the state of O 1s indicated that there are two sorts of oxygen on the surface, the lattice oxygen (O_L_) and the adsorbed oxygen (O_ads_). For O ions in LaFeO_3_, the broad and asymmetric O 1s XPS spectra correspond to two kinds of oxygen chemical states according to the binding energy range. The peak at approximately 529.0 eV is attributed to O_L_ and the other broad peak at around 531.6 eV is attributed to O_ads_, indicating that it is attributed to the contribution of La-O and Fe-O in LaFeO_3_ crystal lattice for the O_L_ signal [34]. For O ions in CeO_2_, Figure 5D shows that the O 1s spectra for the sample contain one band located at 529.1 eV and a shoulder at the higher binding energy of 531.5 eV. The former is originated from lattice oxygen (Ce-O) and the latter can be attributed to the adsorbed oxygen [25]. As for CeO_2_-LaFeO_3_, the O 1s spectra (Figure 5D) also shows a major peak at the 529.3 eV and a broad shoulder peak around 531.5 eV, attributed to the lattice oxygen O^2−^ (La-O, Fe-O and Ce-O) and adsorbed oxygen species. The C1s peaks are presented in Figure 5E.

The atomic concentration ratios were also obtained by XPS. The different atomic concentration ratios on the surface of as-prepared catalysts were calculated and are listed in Table 1. In addition, the Fe/La ratio of LaFeO_3_ (1.2, see Table 1) and CeO_2_-LaFeO_3_ (1.35, see Table 1) are slightly higher than theoretical value indicating that excess amount of iron appears on the surface of LaFeO_3_, which is different from the results of Wei *et al*. [37]. The reason is not clear and needs to be examined further. We suspect that such a result is likely related to the different preparation methods employed compared to those of Wei *et al*.

**Figure 6:**
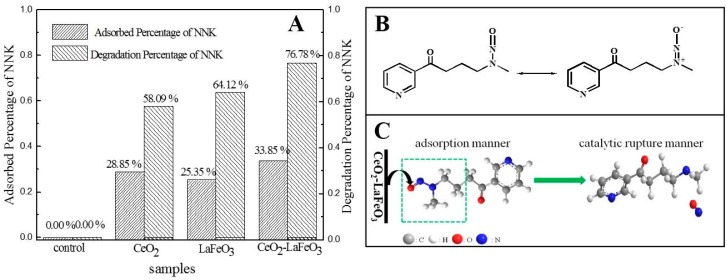
(**A**) Adsorbed and degradation percentage of NNK by CeO_2_, LaFeO_3_ and CeO_2_-LaFeO_3_; (**B**) The resonance structure of NNK; (**C**) The possible adsorption and catalytic rupture manner of NNK on CeO_2_-LaFeO_3_.

The adsorption and catalytic degradation of nitrosamine 4-(methylnitrosamino)-1-(3-pyridyl)-1-butanone (NNK) were investigated to evaluate catalytic activity of CeO_2_-LaFeO_3_. Liquid adsorption results are listed in Figure 6A. Compared with the Blank experiment without any catalyst, the adsorption performance of the three catalysts followed the order of CeO_2_-LaFeO_3_ (33.85%) > CeO_2_ (28.85%) > LaFeO_3_ (23.35%). The degradation of NNK using different catalysts is displayed in Figure 6A. Compared to the blank experiment without any catalyst, the catalytic performance of the three catalysts followed a sequence of CeO_2_-LaFeO_3_ > LaFeO_3_ > CeO_2_. The degradation ratio of NNK is 76.78% for CeO_2_-LaFeO_3_, 58.09% for CeO_2_ and 64.12% for LaFeO_3_. In summary, CeO_2_-LaFeO_3_ composite trapped more NNK (33.85 μg·g^−1^) than CeO_2_ (22.85 μg·g^−1^) and LaFeO_3_ (23.35 μg·g^−1^), despite the former having the smallest specific surface area among the three catalysts. The catalytic activity per surface area of CeO_2_-LaFeO_3_, CeO_2_ and LaFeO_3_ to NNK were tested to be 6.05, 1.23, 2.60 μg·m^−2^, respectively. MCM-22 could adsorb 54% nitrosamines in solution [38], and SBA-15 could catalytically degrade 65% nitrosamines [39]. However, the specific surface of zeolites usually displays 1−2 orders of magnitude higher than perovskite materials. The actual adsorption ability of per m^2^ surface area of CeO_2_-LaFeO_3_ composite is superior to zeolites. What is more, the preparation processes for zeolites consume a large amount of strong acid or base, which is harmful to the environment. Additionally, the high cost of zeolites is a disadvantage for industry application. Conversely, the CeO_2_-LaFeO_3_ composite possesses the advantages of being environmentally friendly, easy to synthesize, inexpensive to prepare, thermally stable and potentially easy to industrialize.

The resonance structure of NNK is shown in Figure 6B and the possible adsorption and degradation mechanism is shown in Figure 6C. NNK possesses the N-N=O functional group, which could be adsorbed on CeO_2_-LaFeO_3_ surface through electrostatic interaction. The catalytic degradation of NNK probably starts from the rupture of the N-N bond in the N-N=O group [39]. Under the same conditions, the CeO_2_-LaFeO_3_ composite showed superior properties to the other two catalysts. This may originate from the synergistic effect of CeO_2_-LaFeO_3_ for degradation of NNK. In the nominal composite CeO_2_-LaFeO_3_ perovskite structure, some Ce^3+^ ions may substitute the La^3+^ ion in the A-site of the LaFeO_3_ perovskite structure. From the XPS result (the Ce^3+^/(Ce^4+^ + Ce^3+^) atomic ratio on the CeO_2_-LaFeO_3_ composite is higher than pure CeO_2_), it was confirmed that the Ce^3+^ ion exists in La_1−x_Ce_x_FeO_3_ crystal phases. Because of the introduction of the Ce^3+^ ion, stronger interactions occur between Fe ions and the adsorbed O_2_. Correspondingly, O_2_ is activated and may lead to higher oxidative reactivity than pure LaFeO_3_ [12], resulting in higher activity for the CeO_2_-LaFeO_3_ composite. Furthermore, CeO_2_ has high oxygen storage capacity, high oxygen mobility and facile reducibility [16]. The synergistic effect of the LaFeO_3_ perovskite structure and CeO_2_ redox property play a crucial role in enhancing the degradation efficiency of NNK. Consequently, the performance for degradation of NNK followed the order of CeO_2_-LaFeO_3_ > LaFeO_3_ > CeO_2_.

## 3. Experimental Section

### 3.1. Materials

4-(methylnitrosamino)-1-(3-pyridyl)-1-butanone (NNK) were purchased from Toronto Research Chemical Inc. (Toronto, ON, Canada). La(NO_3_)_3_·6H_2_O, Ce(NO_3_)_3_·6H_2_O, glucose, urea, Fe(NO_3_)_3_·9H_2_O were purchased from Shanghai Chemical Reagent Company (Shanghai, China). All the chemicals were analytical pure grade and were used as received without further purification. Deionized water was used throughout the experiments.

### 3.2. Synthesis

Based on our previous experiments [40], a typical synthesis method was taken after minor changes: 0.5 M glucose was sealed in a Teflon-lined stainless steel autoclave with 100 mL capacity and maintained at 180 °C for 6 h. After cooling down naturally, the precipitate was harvested by centrifugation and washed thoroughly with deionized water and ethanol. After drying at 60 °C overnight, carbonaceous spheres were obtained. The amounts of 2.18 g La(NO_3_)_3_·6H_2_O and 2.02 g Fe(NO_3_)_3_·9H_2_O were dissolved in 20 mL water. After magnetic stirring for 10 min, 1.00 g carbonaceous spheres were added into the above solution. The solution was subsequently treated ultrasonically at 600 W for 45 min. The resulting solution was aged at room temperature overnight to achieve adsorption-desorption equilibrium between carbonaceous spheres with Fe^3+^ and La^3+^ ions and then centrifuged. After drying at 60 °C overnight, the obtained precursor was denoted as the LaFeO_3_ precursor. The LaFeO_3_ precursor was calcined at 700 °C for 2 h and the LaFeO_3_ was obtained.

Uniform-sized CeCO_3_OH@C nanospheres were synthesized by a hydrothermal method. In a typical synthesis, 0.63 g Ce(NO_3_)_3_·6H_2_O, 5.07 g glucose and 0.86 g urea were dissolved in 100 mL water with magnetic stirring. The solution was transferred into a 100 mL Teflon-lined stainless steel autoclave and hydrothermally treated at 180 °C for 2 h. After cooling down naturally, the precipitate was harvested by centrifugation and washed thoroughly with deionized water and ethanol. After drying at 60 °C overnight, CeCO_3_OH@C was obtained. A certain amount of CeCO_3_OH@C was calcined in a muffle furnace at 550 °C for 2 h to obtain CeO_2_ nanospheres.

**Scheme 1:**
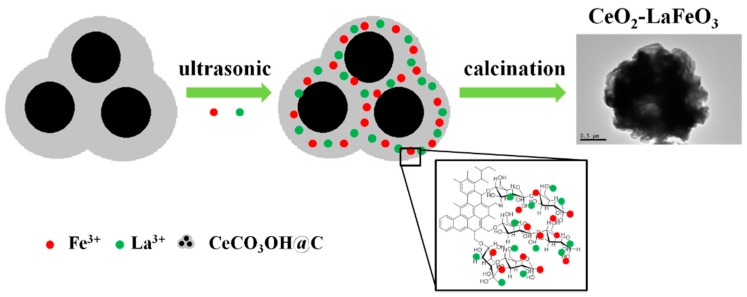
Synthetic route to the CeO_2_-LaFeO_3_ perovskite composite.

In a typical process, 2.18 g La(NO_3_)_3_·6H_2_O and 2.02 g Fe(NO_3_)_3_·9H_2_O were dissolved in 20 mL water with magnetic stirring. Subsequently, 1.00 g CeCO_3_OH@C was added into the above solution and evenly dispersed with the assistance of magnetic stirring. The solution was treated ultrasonically at 600 W for 45 min. The resulting solution was aged at room temperature overnight to achieve adsorption-desorption equilibrium between CeCO_3_OH@C, Fe^3+^ and La^3+^ ions. The solution was centrifuged and washed thoroughly with deionized water and ethanol. The obtained precursor was dried in an oven at 60 °C overnight and denoted as the CeO_2_-LaFeO_3_ precursor. The precursor was calcined at 700 °C for 2 h to obtain CeO_2_-LaFeO_3_ composite. The formation process of CeO_2_-LaFeO_3_ composite is illustrated in Scheme 1. First, La^3+^ and Fe^3+^ are incorporated into CeCO_3_OH@C hydrophilic shell since the surface of CeCO_3_OH@C possesses hydrophilic groups. Second, after calcination, the hydrophilic shell was eliminated and CeO_2_-LaFeO_3_ composite formed. After calcination at 700 °C for 2 h, the spherical structure collapsed and a porous nanostructure formed.

### 3.3. Degradation of 4-(Methylnitrosamino)-1-(3-Pyridyl)-1-Butanone (NNK) by CeO_2_-LaFeO_3_

The catalytic activity for the degradation of NNK by CeO_2_-LaFeO_3_ was performed as follows: 50 mg catalyst was dispersed ultrasonically in 5 mL of 1 μg·mL^−1^ NNK/methanol solution. After that, all of the solutions were evenly dispersed on the same mass quantitative filter papers several times and dried at 30 °C. Quantitative filter papers were made into cigarette shapes and smoked by using a smoking machine. The smoke and the particles after ignition were thoroughly absorbed, extracted, purified, and then transferred to a 25 mL volumetric flask and diluted with methanol to volume. Finally, the diluted methanol solution was analyzed by Agilent 6460 Triple Quad Liquid chromatography-Mass Spectrum (LC/MS, Agilent Technologies, Santa Clara, CA, USA). LaFeO_3_ and CeO_2_ were also tested. As a control experiment, 5 mL of 1 μg·mL^−1^ NNK/methanol solution without catalyst was also tested.

Liquid adsorption of NNK was performed as follows: 20 mg LaFeO_3_, CeO_2_ and CeO_2_-LaFeO_3_ were added into 10 mL of 0.2 μg·mL^−1^ NNK/methanol solution, respectively. The solutions were treated ultrasonically for 1 h and centrifuged at 5000 rpm for 10 min. As a control, 10 mL of 0.2 μg·mL^−1^ NNK/methanol solution without catalyst was also tested. The residual quantity of NNK in absorbed methanol was carried out using Agilent 6460 Triple Quad LC/MS. The mass ratio of catalyst to NNK remained the same (catalyst: NNK = 10 mg:1 μg) in the catalytic and adsorption experiments.

### 3.4. Characterization

X-ray powder diffraction of the as-prepared materials were characterized by X-ray diffraction (XRD, Rigaku D/max-RA, Tokyo, Japan, graphite monochromatized Cu Kα radiation, λ = 1.5406 Å, at 36 kV). FT-IR spectra were recorded with a Nicolet MAGNA-IR 750 instrument (KBr disks, Nicolet Instrument, Madison, WI, USA) in the 4000–400 cm^−1^ regions. Morphologies of samples were examined by field-emission scanning electron microscopy (FE-SEM, Hitachi S4800, Hitachi, Hitachi, Japan). Transmission electron microscopy (TEM) was obtained by a JEM-2100 transmission electron microscope (JEOL, Tokyo, Japan). Thermo-gravimetric analysis and differential scanning calorimetry (TG-DSC) data was recorded with a thermal analysis instrument (WCT-1D, BOIF, Beijing, China) under an airflow atmosphere at the heating rate of 10 °C·min^−1^ from room temperature to 800 °C. Specific surface areas and sorption isotherms of the samples were measured via a nitrogen sorption system at 77 K on a Micromeritics ASAP 2020 analyzer (Norcross, Atlanta, GA, USA). The specific surface area was calculated by the Brunauer-Emmett-Teller (BET) method. The X-ray photoelectron spectra (XPS) were taken on an ESCALab MKII X-ray photoelectron spectrometer (Thermo VG Scientific, West Sussex, UK) to obtain further evidence for the purity and composition of the as-prepared products, using Al Ka radiation as the exciting source.

## 4. Conclusions

In summary, LaFeO_3_ and CeO_2_-LaFeO_3_ porous structured perovskite mixed oxides were successfully synthesized by a novel surface-ion adsorption method using carbonaceous microspheres and CeCO_3_OH@C as templates. The surface-ion adsorption method could be a promising method to synthesize various perovskite composite materials. The performance for degradation of 4-(methylnitrosamino)-1-(3-pyridyl)-1-butanone (NNK) followed a sequence of CeO_2_-LaFeO_3_ > LaFeO_3_ > CeO_2_. The CeO_2_-LaFeO_3_ composite exhibited excellent catalytic activity for NNK degradation, making it a promising candidate for environmentally friendly applications. Based on these results, other catalysts with structures similar to CeO_2_-LaFeO_3_ may also be used for the degradation of tobacco-specific nitrosamines (TSNAs) such as NNK in tobacco. Furthermore, the investigations of other Ce-doped perovskite materials, such as CeO_2_-LaCoO_3_ and CeO_2_-LaNiO_3_ for the degradation of NNK, are in progress.

## Figures and Tables

**Table 1:** Surface elemental composition/at % of as-prepared catalysts obtained from XPS.

Samples	Fe	Ce	La	O	C
CeO_2_	-	22.14	-	52.01	25.85
LaFeO_3_	17.01	-	14.19	51.6	17.2
CeO_2_-LaFeO_3_	13.58	9.3	10.1	49.47	17.55
